# Nonmetric sex estimation in a contemporary Indonesian population: a validation study using clinical pelvic MSCT scans

**DOI:** 10.1007/s00414-024-03266-4

**Published:** 2024-06-12

**Authors:** Ridhwan Lye, Zuzana Obertová, Nur Amelia Bachtiar, Daniel Franklin

**Affiliations:** 1 Centre for Forensic Anthropology, M420, The University of Western Australia, Crawley, WA 6009 Australia; 2 Radiology Department, Hasanuddin University, Jalan Perintis Kemerdekaan KM. 10, Talamanrea, 90254 Makassar Indonesia

**Keywords:** Forensic anthropology, Sexual dimorphism, Sex estimation, Pelvic nonmetric traits, Logistic regression, Indonesia

## Abstract

Klales et al. (2012) is a popular standard for the estimation of skeletal sex. Since its publication, a number of studies have demonstrated that population-specific applications of Klales improve classification accuracy. However, it has also been shown that age appears to affect the expression of dimorphism in the pelvis across the lifespan. As such, the present study examines the accuracy of Klales, and the modified global standard of Kenyhercz et al. (2017), in a contemporary Indonesian population, including quantifying the effect of age. Pelvic multi-slice CT scans of 378 individuals (213 female; 165 male) were analysed in *OsiriX*®. Both standards were tested and Indonesian-specific models thereafter derived.

When applied to the Indonesian sample, both the Klales and Kenyhercz standards resulted in lower classification accuracy relative to the original studies. In considering the Indonesian-specific models, the ventral arc was the most accurate for the classification of sex, at 93.3% with a − 3.0% sex bias. The accuracy of the three-trait model was 94.4%, with a − 5.5% sex bias. Age was shown to significantly affect the distribution of pelvic trait scores. As such, age-dependent models were also derived, with the standard for individuals between 30 and 49 years the most accurate, at 93.1% and a sex bias of − 4.0%. Accuracy was lower in individuals aged ≥ 50 years, at 91.3% and a sex bias of 4.1%. These findings support the importance of establishing population-specific standards and to facilitate improved accuracy and capabilities for forensic practitioners in Indonesia.

## Introduction

The pelvic complex is the most dimorphic within the human skeleton; accordingly, this region is amongst the most studied in a forensic anthropological context relative to the estimation of skeletal sex. The three pelvic traits described in Phenice [1] – the ventral arc, medial aspect of the ischiopubic ramus, and subpubic concavity – are the most commonly used amongst forensic practitioners to supply an estimation based on a morphoscopic assessment [2]. This is largely because the latter approach is relatively simple and quick to deploy and requires no specialised equipment. Such assessments can also be applied to fragmentary remains, unlike their morphometric counterparts [2, 3].

Despite the popularity of the Phenice standard, a lack of quantifiable measures of error (e.g., probability values associated with sex classification) do not meet contemporary evidentiary requirements for medicolegal casework [4]. Klales et al. [5] improves the Phenice standard by expanding the binary absent/present assignment for each trait to a 5-point ordinal scale, thus representing more variation in trait morphology. By applying binary logistic regression (BLR), their study also introduced probabilities associated with sex classification, and weighted traits based on their discriminatory value.

The Klales et al. [5] standard has been applied across population groups to assess its appropriateness for use outside the United States (US). In a Mexican population, Gómez-Valdés et al. [6] reported classification accuracies of 100% for females and 90% for males. Oikonomopoulou et al. [7] reported classification accuracies of 98.82% for females and 89.52% for males in a Greek population. Kenyhercz et al. [8], testing on six different population groups, including Caucasian and African American, Caucasian and Black South Africans, unidentified Hispanic individuals crossing through the Mexico-US border, and Thai, reported classification accuracies between 91.7% and 100.0% for females and 82.3% and 95.6% for males. Also included in their study were accuracy statistics with all six population groups combined (i.e., a global function): 94.8% for females and 96.8% for males. In all of those studies, the level of classification accuracy achieved was higher than those reported in Klales et al. [5] (98.0% female; 74.4% for male).

At present, Klales et al. [5] has not been extensively validated for use with clinical digital imaging, such as computed tomography (CT) scans. Colman et al. [9] surmised that the use of CT scans was viable as a proxy to physical skeletal material, reporting Cohen’s Kappa values between 0.74 and 0.82 when assessing agreement of scoring between physical material and its digital counterpart. However, the authors acknowledge the lack of validation studies testing the ordinal scoring system on CT scans. Johnstone-Belford et al. [10] tested the original Phenice [1] standard in CT scans from an Australian population, reporting classification accuracies of 97.3% for females and 87.6% for males. The application of traditional morphoscopic standards in CT scans would thus serve to further validate it as an alternative to physical skeletal material [11].

Though research into age-related changes in pelvic morphology as a whole have been undertaken [12], age-related changes in the three pelvic traits have not been extensively assessed [13]. In other regions of the pelvis, such as the greater sciatic notch, it has been shown that the trait shifts to be more masculine (i.e., a narrower greater sciatic notch) with increasing age. This trend is more apparent in females, which resulted in increased misclassification from age 50 onwards [14, 15]. Age-related changes also appear to affect the morphology of the ventral arc, with its surface becoming more irregular with increasing age, although such assertions have not been empirically tested [16].

There is a paucity of sex estimation standards appropriate for application in the Southeast Asian region; researchers have endeavoured to address this by developing population-specific standards with other bones, such as the skull [17, 18]. Within the context of forensic anthropological research in Indonesia, the non-invasive nature of post-mortem CT scanning accords with cultural and religious considerations for the handling of the deceased [19, 20]. As the country has also recorded a high number of mass fatality events [21], the use of CT scanning will enable its dissemination to forensic practitioners for both disaster victim identification and domestic casework, increasing productivity and ultimately serving to improve the likelihood of achieving positive identifications [22].

In recognising the importance of validating standards on their respective populations, and with few validation studies using CT scans, the objective of this present study is to evaluate the accuracy of Klales et al. [5] and the global function in Kenyhercz et al. [8] in an Indonesian population. Specifically, this study will aim to develop forensically applicable predictive models for the estimation of skeletal sex in the contemporary Indonesian population and to statistically quantify the effect of age on the latter data.

## Materials and methods

### Study sample

Clinical pelvic multi-slice CT (MSCT) scans were obtained from the Picture Archiving and Communications System database at the Dr Wahidin Sudiohusodo General Hospital (RSWS), Hasanuddin University, Makassar. Imaging was performed with a Siemens Healthineers SOMATOM go.Top 128-slice CT scanner, with slice thickness between 1.0 and 1.5 mm (96.0% of all scans are 1.5 mm). Scans represent patients who attended RSWS for clinical evaluation between January 2020 and August 2022; all scans are anonymised prior to receipt, except for recorded sex and age. Those scans which present pathology and/or other abnormalities that would obscure the visualisation and scoring of pelvic traits were excluded at this stage.

**Fig. 1:**
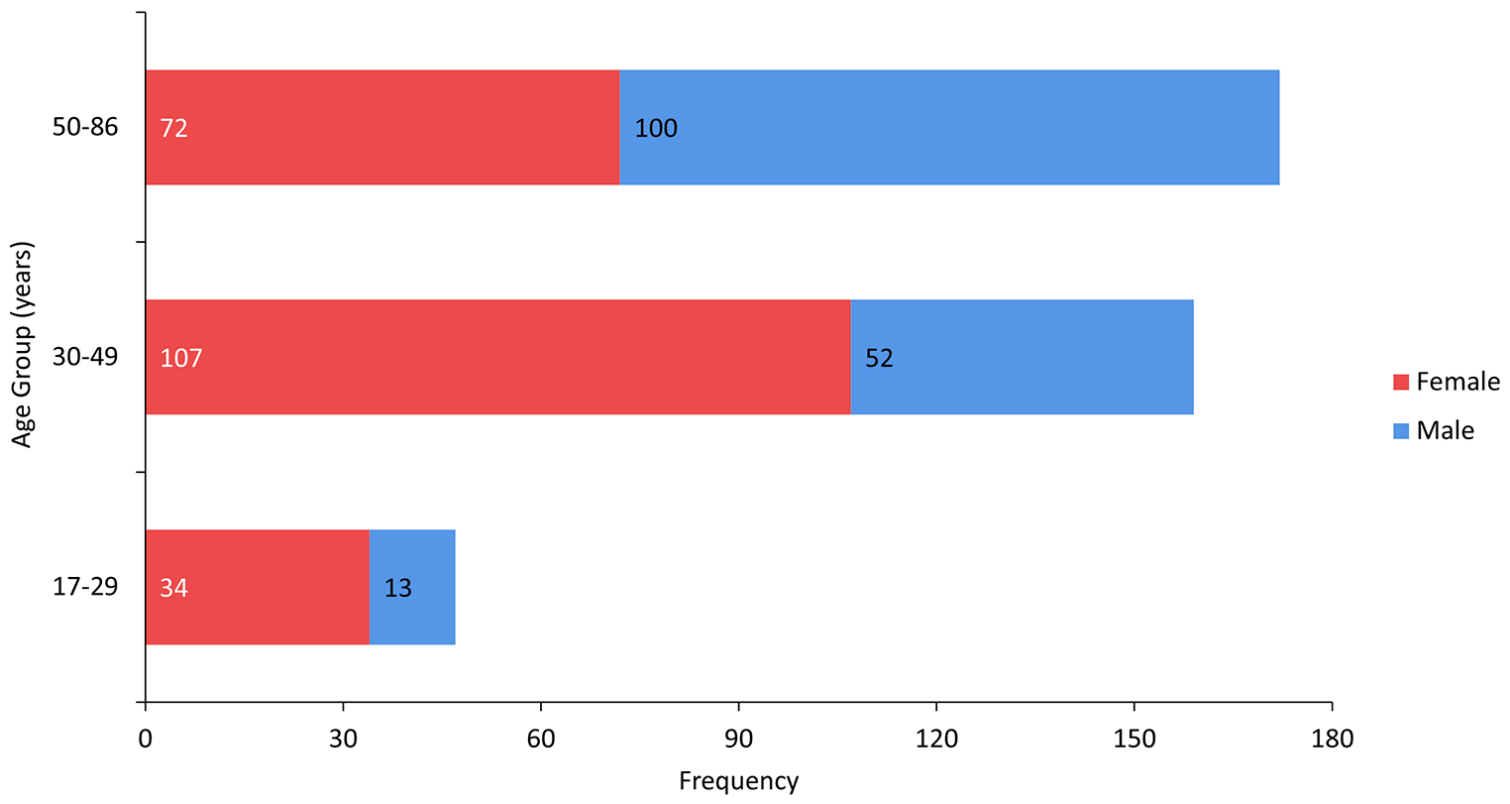
Distribution of the Indonesian sample by sex and age group with frequencies in each group indicated

In total, 378 scans were analysed, comprising 213 female and 165 male individuals. The stated age range was 17 to 86 years: (female mean = 43.7 years, SD = 12.8 years; male mean = 50.9 years, SD = 13.3 years). The sex and age distribution of the Indonesian sample is shown in Fig. 1.

Approvals for this study were obtained from the Human Ethics Committee of the Office of Research at the University of Western Australia (2021/ET000377) and the Office of the Director-General of Health Sciences from the Ministry of Health, Republic of Indonesia, through Hasanuddin University (LB.02.01/2.2/6807/2022).

### Visualisation and assessment

**Fig. 2:**
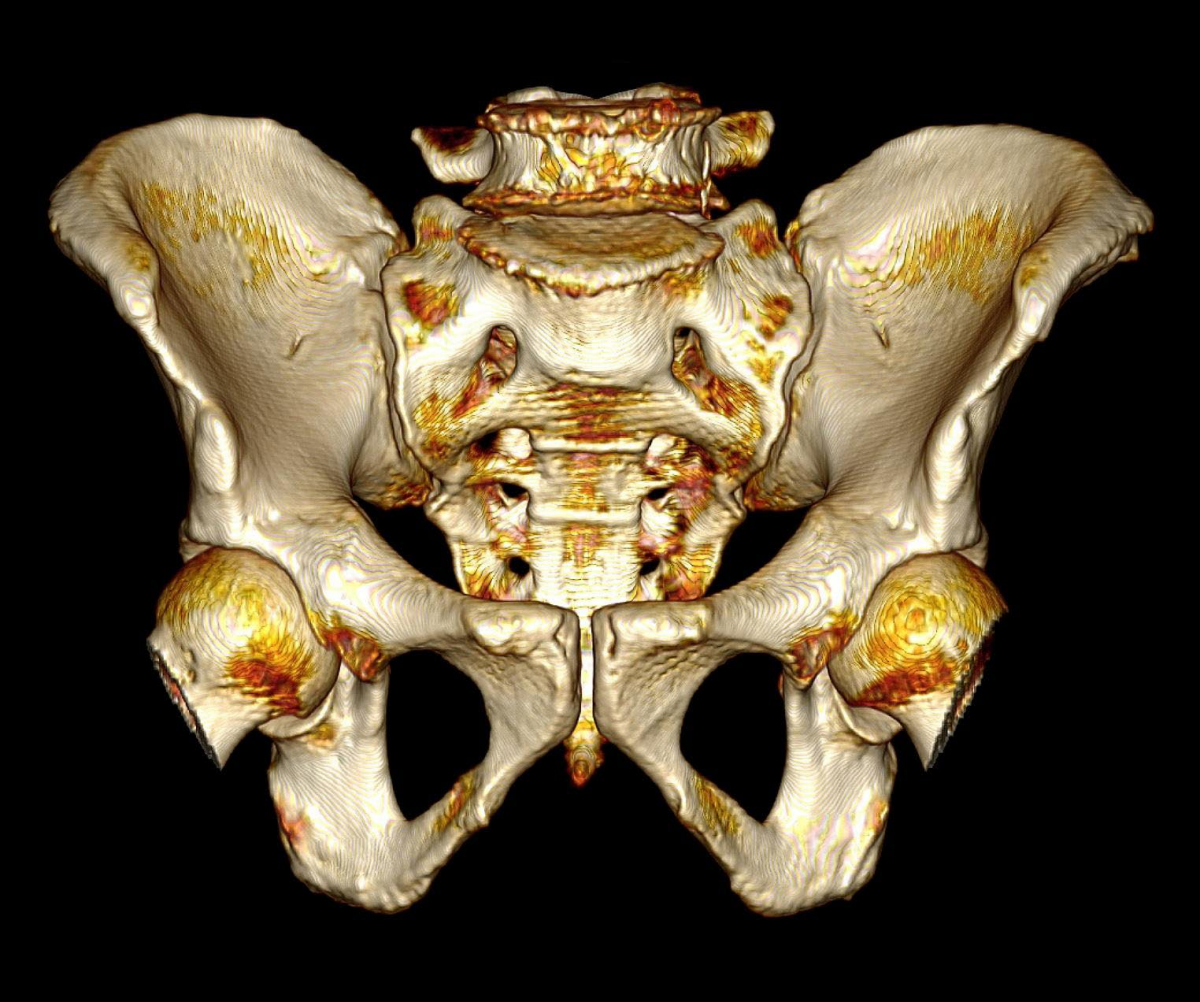
Three-dimensional volumetric reconstruction showing an anterior visualisation of an isolated male pelvis from the Indonesian sample

*OsiriX*® *v13.0.1* was used for the visualisation and three-dimensional (3D) volumetric reconstruction of all MSCT scans. The ‘3D rotate’ and ‘pan’ function were used to orientate each scan for assessment and scoring; an example is shown in Fig. 2. The ‘High Contract’ 3D preset was used in the volume rendering window. CLUT was set to ‘VR Muscles-Bones’, and no convolution filters were applied.

**Fig. 3:**
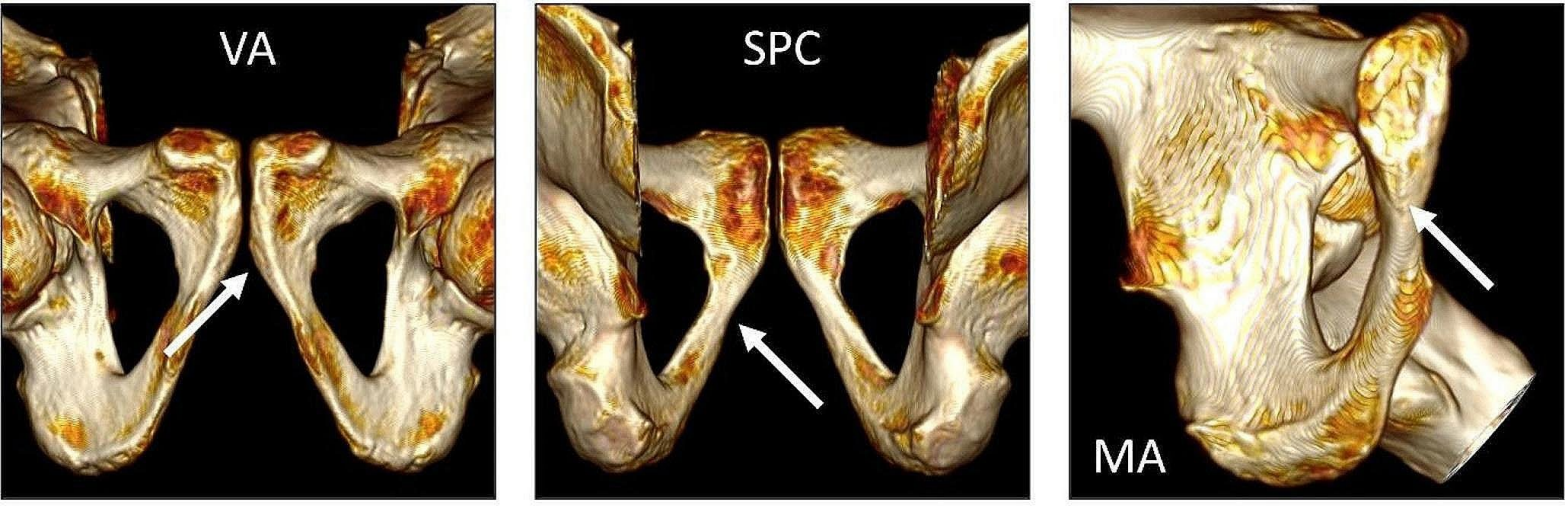
The three pelvic traits used in the Klales et al. [5] standard visualised in a 3D environment: ventral arc (VA), subpubic concavity (SPC), and the medial aspect of the ischiopubic ramus (MA)

The assessment and scoring of the ventral arc (VA), subpubic concavity (SPC), and medial aspect of the ischiopubic ramus (MA), were performed following the written descriptions and visual illustrations of Klales et al. [5]. Traits were scored on a five-point ordinal scale from 1 to 5. No assumptions related to masculinity or femininity were associated with any of the scores, and both the left and right innominate were scored. Examples of these traits as visualised in the 3D platform are provided in Fig. 3.

### Statistical analysis

*IBM SPSS Statistics v29.0.0* was used for all statistical analyses in this study.

#### Intra-observer agreement

A random subset of 50 MSCT scans of the left innominate were assessed to quantify intra-observer agreement. In this subset, 25 female (mean age = 48.9 years; SD = 16.7 years) and 25 male scans (mean age = 49.4 years; SD = 15.4 years) were included. These scans were blinded (e.g., no demographic data provided) and assessed by the lead author (RL) three times, with each repeat assessment performed with an interval of at least 24 h. Intraclass correlation coefficient (ICC) was used to calculate and interpret agreement as follows: poor (< 0.50); moderate (0.50 to 0.75); good (0.75 to 0.90); and excellent agreement (> 0.90) [23].

#### Bilateral asymmetry

Bilateral asymmetry is quantified to facilitate statistical interpretation of variances in scoring between the left and right innominate, and thus determine if those differences imply that side-specific prediction models are required. Data are tested using a Wilcoxon signed-rank test (*Z*).

#### Trait score distributions

Pelvic trait score distributions are calculated and reported by sex. Differences in the distributions between females and males are assessed using a Mann-Whitney U test. These trait frequency values are also used to derive the probability of obtaining a specific score for a trait. This probability is derived from the proportionality of score assigned for that trait against the distribution and uses the following equation:

$$pf=\frac{\text{\%} \text{f}\text{e}\text{m}\text{a}\text{l}\text{e}}{\text{\%} \text{f}\text{e}\text{m}\text{a}\text{l}\text{e}+\text{\%} \text{m}\text{a}\text{l}\text{e}}$$

where *% female* and *% male* are the proportions of females and males assigned a specific score on a trait. The probability of an individual classified as male is calculated following this equation:

$$pm=1-pf$$

If *pf* > 0.50, the individual is likely female; if *pm* > 0.50, the individual is likely male.

#### Effect of age on trait score distributions

To assess whether age had an influence in pelvic trait distributions, the sample in the present study was split into three age groups: < 30 years; 30–49 years; and ≥ 50 years (see Fig. 1). Kruskal-Wallis tests (*H*) are then performed separately for females and males. When significant differences are reported, post-hoc testing with Bonferroni corrections are used to determine which age bracket pairs have statistically significant differences in trait score distributions.

#### Validation of the Klales et al. (2012) and Kenyhercz et al. (2017) functions

The three-trait function described in Klales et al. [5] as applied to the Indonesian sample is tested to serve as a comparison against its performance with the original publication, given that the function was derived from a sample of individuals from the US. The BLR function used in Klales et al. [5] is provided below:

$$2.726\left(\text{V}\text{A}\right)+1.073\left(\text{S}\text{P}\text{C}\right)+1.214\left(\text{M}\text{A}\right)-16.312$$

The global pooled-population function described in Kenyhercz et al. [8] is tested in the Indonesian sample to assess accuracy and its suitability for forensic application and is provided below:

$$1.4296\left(\text{V}\text{A}\right)+1.0415\left(\text{S}\text{P}\text{C}\right)+0.9752\left(\text{M}\text{A}\right)-10.0139$$

Differences in performance are calculated based on the comparisons of classification accuracy and sex bias values for the two functions. For this study, sex bias is calculated as the difference between male and female classification accuracy. Positive sex bias values indicate disproportionate male misclassification, while negative values indicate female misclassification.

#### Univariate and multivariate models for the Indonesian population

Each individual trait is used as an independent variable when deriving univariate predictive models. All possible two-trait pairings, and a three-trait combination, are used as independent variables in multivariate predictive models. To derive these models, approximately 75% of the sample (*n* = 288) was randomly assigned for training, while the remaining 25% is used for validation (i.e., hold-out; *n* = 90). Sex is coded as 0 for female and 1 for male. Sex-specific and total classification accuracy, and sex bias values, for both the training and validation samples, are provided. Traits that significantly contributed to the model fit (α < 0.05) are indicated by their associated Wald statistic (*W*).

#### Age-dependent multivariate models for the Indonesian population

As detailed above, there is some evidence to suggest that pelvic traits trend toward higher scores as an individual ages. Ascertaining a specific age-at-death of an unknown adult individual is inherently inaccurate, thus an age range is instead more appropriately reported. Accordingly, the three age groups (AG) as indicated in Fig. 1 are used. Facilitating the derivation of predictive models when considering age-at-death was undertaken in two ways. The first was using AG as an additional independent variable, along with the three pelvic traits assessed. The second was assessing each AG as individual subgroups (i.e., perform a subgroup analysis deriving models of each AG) to derive their respective models. Nagelkerke *R*^2^ values are used to indicate the proportion of variance in sex estimation that is explained by each age-dependent model.

## Results

### Intra-observer agreement

The ICC estimates and 95% confidence interval (CI) for intra-observer agreement on each pelvic trait is based on a single-rater, absolute-agreement, 2-way mixed-effect model. Good agreements were reported for the ventral arc (ICC = 0.823, 95% CI [0.736, 0.888]) and subpubic concavity (ICC = 0.851, 95% CI [0.775, 0.907]). The medial aspect had moderate agreement (ICC = 0.581, 95% CI [0.425, 0.717]).

### Bilateral asymmetry

Wilcoxon signed-rank tests for all traits reported significant differences in scoring between the left and right innominate for the ventral arc only, *Z* = − 2.84, *p* = .005. Non-significant differences were reported for the subpubic concavity, *Z* = 0.47, *p* = .642, and the medial aspect, *Z* = − 0.29, *p* = .774. Given that two of the three pelvic traits reported non-significant differences in scoring between both innominate bones, predictive models derived from the Indonesian sample in this study will only include scores recorded from the left innominate.

### Trait score distributions

Table 1 shows the distribution of pelvic trait scores for the Indonesian sample. All character states were expressed for all pelvic traits observed. Differences in trait score distributions were significant for all traits between females and males: ventral arc, *U*(2) = 14.24, *p* < .001; subpubic concavity, *U*(2) = 11.65, *p* < .001; and the medial aspect, *U*(2) = 10.26, *p* < .001. In general, females tend to have lower pelvic trait scores, and *vice versa* for males. Females were also noted to have a wider variation of scores relative to males. Probability values associated with each character state for all pelvic traits are provided in Table 2.

**Table 1:** Frequency distributions of pelvic trait scores sorted by sex

Pelvic Trait^a^	Score Frequency				
	1 *n* (%)	2 *n* (%)	3 *n* (%)	4 *n* (%)	5 *n* (%)
VA					
Female	45 (21.1)	91 (42.7)	53 (24.9)	18 (8.5)	6 (2.8)
Male	1 (0.6)	6 (3.6)	19 (11.5)	52 (31.5)	87 (52.7)
SPC					
Female	31 (14.6)	96 (45.1)	75 (35.2)	8 (3.8)	3 (1.4)
Male	2 (1.2)	13 (7.9)	68 (41.2)	48 (29.1)	34 (20.6)
MA					
Female	33 (15.5)	84 (39.4)	75 (35.2)	17 (8.0)	4 (1.9)
Male	7 (4.2)	11 (6.7)	59 (35.8)	60 (36.4)	28 (17.0)

^a^ VA = ventral arc; SPC = subpubic concavity; MA = medial aspect of the ischiopubic ramus

**Table 2:** Probability values based on assigned pelvic trait scores. See Table 1 for trait score frequency distributions

Pelvic Trait^a^	Score Probabilities				
	1	2	3	4	5
VA					
Female	0.972	0.922	0.684	0.211	0.051
Male	0.028	0.078	0.316	0.789	0.949
SPC					
Female	0.923	0.851	0.461	0.114	0.064
Male	0.077	0.149	0.539	0.886	0.936
MA					
Female	0.785	0.855	0.496	0.180	0.100
Male	0.215	0.145	0.504	0.820	0.900

^a^ VA = ventral arc; SPC = subpubic concavity; MA = medial aspect of the ischiopubic ramus

From Table 2, it was observed that ventral arc trait scores from 1 to 3 were more likely associated with female sex, while scores from 4 to 5 were likely associated with male sex. Subpubic concavity scores from 1 to 2 were more likely associated with female sex and scores from 3 to 5 were noted for male sex. The medial aspect had three divisions: scores from 1 to 2 were associated with female sex, a score of 3 was designated as indeterminate, and scores from 4 to 5 were associated with male sex. The indeterminate designation was considered as the probability values were at, or approaching, chance.

### Effect of age on trait score distributions

Interpretation of the Kruskal-Wallis test data indicated a significant effect of age in pelvic trait distributions in both females and males. Specifically, the ventral arc and medial aspect distributions were significantly different in females, *H*(2) = 7.76, *p* = .021; and *H*(2) = 11.17, *p* = .004, respectively. In males, only the medial aspect distribution was significantly different, *H*(2) = 7.96, *p* = .019. Post-hoc testing using Bonferroni correction demonstrated differences in the < 30 and ≥ 50 year groups, in both female ventral arc and medial aspect distributions, *p* = .016 and *p* = .003, respectively. In males, significant differences were reported in the < 30 year and 30–49 year groups for the medial aspect, *p* = .014.

### Validation of the Klales et al. (2012) and Kenyhercz et al. (2017) functions

**Fig. 4:**
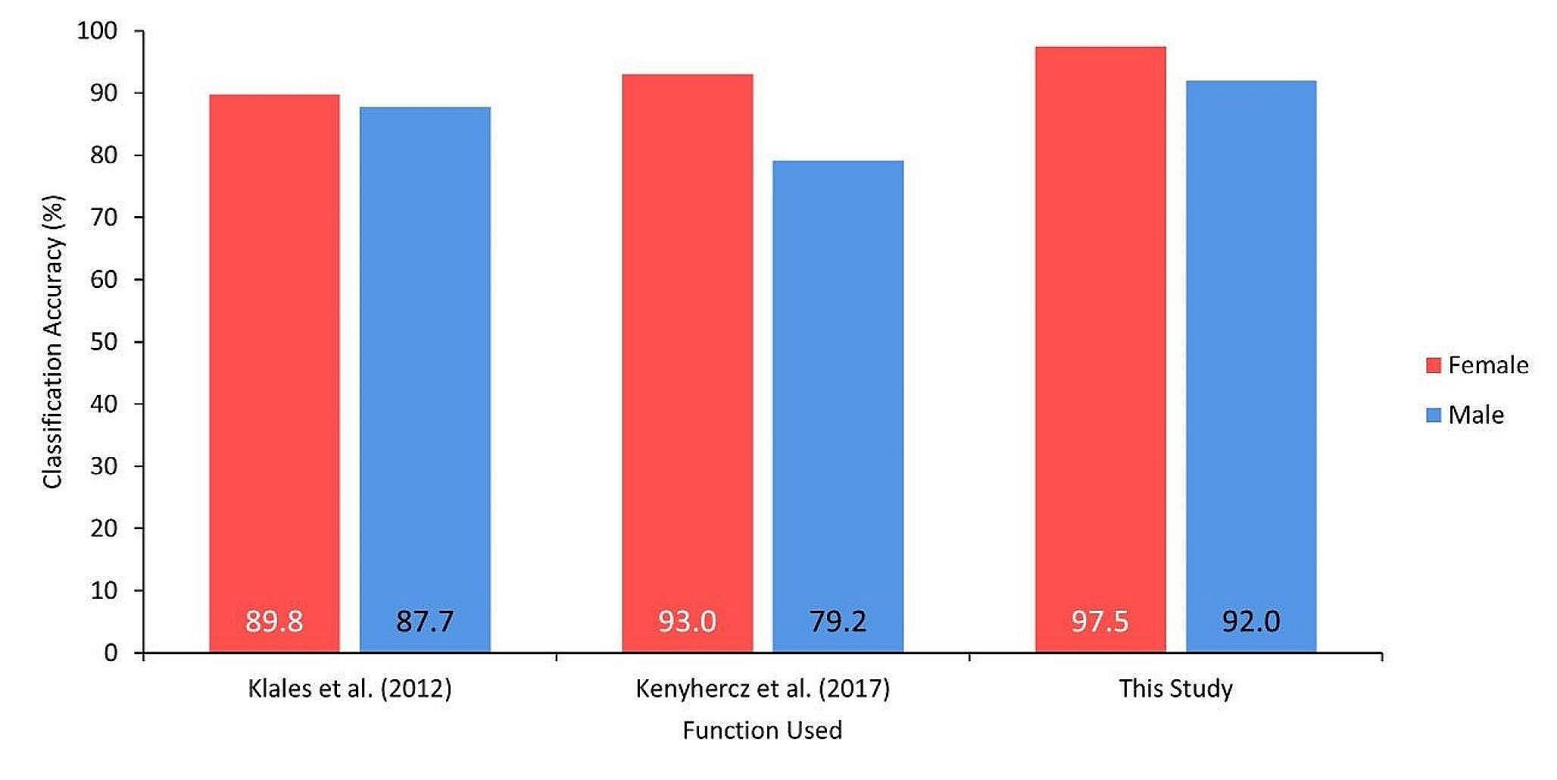
Sex-specific classification accuracies derived from the three-trait functions in Klales et al. [5], Kenyhercz et al. [8], and this study, when applied to the Indonesian sample

The three-trait function in Klales et al. [5] as applied to the Indonesian sample resulted in correct classification by sex of 89.8% for females and 87.7% for males (sex bias: – 2.1%). The pooled global population function in Kenyhercz et al. [8] as applied to the Indonesian sample resulted in classification accuracy of 93.0% for females and 79.2% for males (sex bias: − 13.8%). These results are presented in Fig. 4.

### Univariate Indonesia-specific models

Three univariate models (S1 to S3) were derived for each pelvic trait and are detailed in Table 3. In the training subset, Function S1 (ventral arc) was most accurate, at 87.3% for females and 80.9% for males. Function S3 (medial aspect) was least accurate, at 90.8% for females and 51.3% for males. The sex bias was smallest in Function S1, with a value of − 6.4%, while Function S2 (subpubic concavity) had the largest value, at 42.6%. In the validation (i.e., hold-out) subset, Function S1 was again most accurate, at 95.0% for females and 92.0% for males (sex bias: − 3.0%). Function S2 was least accurate, at 95.0% for females and 44.0% for males (sex bias: − 51.0%).

**Table 3:** Indonesia-specific predictive models, their associated training and validation classification accuracies, along with sex bias

Predictive Model^a^		Training Accuracy (%)					Validation Accuracy (%)			Bias (%)
		Female	Male	Total	Bias (%)		Female	Male	Total	
Univariate										
S1	2.066(VA) – 7.104	87.3	80.9	84.7	–6.4		95.0	92.0	93.3	–3.0
S2	1.736(SPC) – 5.304	94.8	52.2	77.8	–42.6		95.0	44.0	66.7	–51.0
S3	1.182(MA) – 3.750	90.8	51.3	75.0	–39.5		87.5	58.0	71.1	–29.5
Multivariate										
M1	1.922(VA) + 0.727(MA) – 8.724	90.8	84.3	88.2	–6.5		90.0	90.0	90.0	0.0
M2	1.733(VA) + 0.873(SPC) – 8.564	87.9	80.0	84.7	–7.9		97.5	92.0	94.4	–5.5
M3	1.543(SPC) + 0.968(MA) – 7.621	91.3	74.8	84.7	–16.5		95.0	70.0	81.1	–25.0
M4	1.631(VA) + 0.718(MA) + 0.846(SPC) – 10.231	91.3	85.2	88.9	–6.1		97.5	92.0	94.4	–5.5

^a^ VA = ventral arc; SPC = subpubic concavity; MA = medial aspect of the ischiopubic ramus

### Multivariate Indonesia-specific models

Four multivariate models (M1 to M4) were derived from all possible two-trait, and a three-trait, combination (Table 3). All pelvic traits significantly contributed to every multivariate model, *W*(1) ≥ 8.24, *p* ≤ .004. In the training subset, the most accurate two-trait combination was Function M1 (ventral arc and medial aspect), with a classification accuracy of 90.8% for females and 84.3% for males (sex bias: − 6.5%). The least accurate two-trait combination was Function M3 (subpubic concavity and medial aspect), with a classification accuracy of 91.3% for females and 74.8% for males (sex bias: − 16.5%). Function M4, incorporating all pelvic traits, had a classification accuracy of 91.3% for females and 85.2% for males (sex bias − 6.1%). In the validation subset, Function M1 was also most accurate, at 90.0% for both females and males. Function M3 was least accurate, with a classification accuracy of 95.0% for females and 70.0% for males (sex bias: − 25.0%). Function M4 had a classification accuracy of 97.5% for females and 92.0% for males (sex bias: − 5.5%).

### Age-dependent models

The effect of age on pelvic trait distribution in the Indonesian sample were noted previously in this section. As such, models utilising age as an additional independent variable (Function A1) and models for each specific age group (Functions A2 to A4) were derived and are described in Table 4. Function A1, which included age groups (AG) as an independent variable, correctly classified 93.4% of females and 87.9% of males (sex bias: − 5.5%). AG significantly contributed to the model fit, *W*(1) = 12.61, *p* < .001.

**Table 4:** Classification accuracies and sex biases associated with predictive models when age is considered

Predictive Model^a^		Accuracy (%)			Bias (%)
		Female	Male	Total	
Age as Independent Variable					
A1	1.440(VA) + 0.616(SPC) + 0.647(MA)	93.4	87.9	91.0	–5.5
	+ 0.917(AG) – 10.888				
< 30 years					
A2	0.764(VA) + 0.484(SPC) – 0.274(MA)	91.2	38.5	76.6	–52.7
	– 4.026				
30–49 years					
A3	1.865(VA) + 1.034(SPC) + 1.457(MA)	94.4	90.4	93.1	–4.0
	– 14.686				
≥ 50 years					
A4	1.463(VA) + 0.625(SPC) + 0.540(MA)	88.9	93.0	91.3	4.1
	– 7.753				

^a^ VA = ventral arc; SPC = subpubic concavity; MA = medial aspect of the ischiopubic ramus; AG = age group

Function A2 (< 30 years) was least accurate, at 91.2% for females and 38.5% for males (sex bias: − 52.7%). The medial aspect was weighted inversely to the other two pelvic traits and had the lowest coefficient of determination, Nagelkerke *R*^2^ = 0.289. Function A3 (30–49 years) was most accurate, at 94.4% for females and 90.4% for males (sex bias: − 4.0%); it also had the highest coefficient of determination, Nagelkerke *R*^2^ = 0.816. Classification accuracy was reduced slightly in Function A4 (≥ 50 years), at 88.9% for females and 93.0% for males (sex bias: 4.1%).

## Discussion

The aim of this present study was to assess the applicability of the Klales et al. [5] standard and global function in Kenyhercz et al. [8] relative to application in an Indonesian population. This study also derived Indonesia-specific models to serve as a basis for comparison against the two aforementioned models. Age was also reported to have a significant effect on the distribution of pelvic trait scores.

### Observer agreement

Intra-observer agreement was good for both the ventral arc and subpubic concavity, while the medial aspect had moderate agreement. This is in contrast with Klales et al. [5], where the subpubic concavity had the lowest agreement. Furthermore, no consistent trend (e.g., whether the same trait had consistently the highest agreement) was observed across studies reporting intra-observer agreement [7, 9, 24]. Inter-observer agreement tests were not considered as multiple studies have reported agreement scores for different observers in the literature [5, 7, 9, 24–26].

The low intra-observer agreement in the medial aspect may likely be due to the limitation of trait assessment with CT scans. The ascending ramus ridge described in Klales et al. [5] may require some tactile feedback to discern between its various character states: ‘narrow’, ‘medium’ and ‘very broad’. Braun et al. [26] noted that this lack of tactile feedback may result in inconsistent scoring of that trait. Unfortunately, no other study has attempted to modify descriptors in Klales et al. [5] to address the lack of tactile feedback in scoring the medial aspect in a 3D modality. A number of studies have assessed the differences in visualising pelvic traits in 3D reconstructed images. Braun et al. [26] indicated scoring with Klales et al. [5] between both physical and digital skeletal material had moderate observer agreement. Likewise, Colman et al. [9] reported similar results when scoring with Klales et al. [5], suggesting that the use of CT scans could be used as a viable alternative to physical skeletal material.

### Inter-population variation in pelvic trait expression

A visual comparison of the three-trait functions from Klales et al. [5], Kenyhercz et al. [8], and the Indonesia-specific model derived in this study is shown in Fig. 4. The three-trait Indonesia-specific function was more accurate than the original and global function. When the Klales et al. [5] three-trait function was applied to the Indonesian sample, differences were noted in both female and male classification accuracies to its original values, with female accuracy dropping by 8.2% and increasing by 13.3% for males, closing the sex bias to − 2.1% from its original value of − 23.6%. The pooled-population three-trait function in Kenyhercz et al. [8] performed poorly in the Indonesian sample, with female accuracy dropping by 1.8% and by 17.6% for males. These results widened sex bias to − 13.8%, from − 2.0% in the original publication. In all three functions, males were misclassified (i.e., female sex bias).

The derivation of Indonesia-specific models improved classification accuracy, which is in line with other studies testing the Klales et al. [5] standard on their respective population groups [6, 7, 27]. Kenyhercz et al. [8] suggest that the population origin of an unknown individual need not be considered in the context of routine casework, which is mitigated by the application of a ‘global function’. However, the addition of Indonesia-specific models in this study is justified, as classification accuracy was higher. The pelvic trait distributions also provide meaningful comparisons with other population groups, which can further discussions on the effect of intrinsic and extrinsic factors that influence sex-based differences in pelvic trait expressions between population groups [28–30].

### Indonesia-specific models

In total, seven models – three univariate and four multivariate – were derived from the Indonesian sample, as detailed in Table 3. The ventral arc was the most accurate in the validation subset, with an overall classification accuracy of 93.3% and a sex bias of − 3.0%. As all other univariate functions had overall classification accuracies below 85.0%, and sex bias values outside of acceptable limits (i.e., absolute difference greater than 5.0%), these functions should not be used in forensic practice. Likewise, univariate assessments using the ventral arc is not recommended.

Multivariate models with all possible trait combinations are presented in this study. This is in contrast to much of the extant published literature, which feature only the three-trait function [5, 6, 8]. Validation classification accuracies ranged from 90.0 to 97.5% in females, and from 70.0 to 92.0% in males. Function M1, incorporating the ventral arc and medial aspect, was the most accurate two-trait model, with an overall classification accuracy of 90.0% and no sex bias. The three-trait model, Function M4, had an overall classification accuracy of 94.4% and a sex bias of − 5.5%. Only Function M3 had an overall classification accuracy below 85.0% and sex biases outside of acceptable limits, indicating that it should not be applied in forensic practice. However, as all two-trait combinations report statistically significant Wald values for their respective model fits, Functions M1 to M3 may be considered ‘best-fits’, despite the subpar performance of Function M3 relative to M1 and M2.

### Effect of age

Although results from this study report lower overall classification accuracies between Functions A3 and A4 in Table 4, its difference is only 1.8%; both functions still maintain overall classification accuracies above 85.0% and an absolute sex bias of less than 5.0%, making them suitable for forensic application. Sex bias was also shifted from overestimating females in Function A3, to overestimating males in Function A4. This change is consistent with studies that report a shift toward more masculine presentations with pelvic traits in individuals aged 40 and older, and that such changes are more commonly observed in females [14, 15, 25]. These age-related changes may result in increased female misclassifications (i.e., greater male sex bias).

The medial aspect weighting in Function A2 was negative, while its weighting in all other functions were positive. This inversion is noteworthy as the trend suggests higher trait scores are more likely to be associated with individuals of male sex. When examining the composition of the sample subset aged < 30 years, the mean medial aspect trait scores in females and males were 2.82 and 2.77, respectively. These values may have influenced the inverse weighting in Function A2, as the mean score for females is higher than males. No other study utilising this standard has reported a pelvic trait variable with an inversed weighting.

In contrast to the age-independent models, Wald values for both the medial aspect and subpubic concavity in Function A2 were non-significant. These suggest that the inclusion of these traits in Function A2 did not significantly contribute to the model fit. This is also reflected in its low Nagelkerke *R*^2^ value, which indicate that only 28.4% of variance in sex estimation is explained by Function A2. This, coupled with the small sample size in its associated subset (*n* = 47), suggest that Function A2 cannot be reliably applied in forensic practice.

### Study limitations

Indonesia is a country with a diverse population mix spanning over 17,000 islands [31]. The results from this study capture only a minute subset of this population. Future studies that include samples taken from other Indonesian subpopulations would serve to improve and further validate the statistical models described in this study. Such endeavours have already been noted in other countries with large and disparate population centres, with results indicating overall classification accuracy above 90.0% (e.g [32]).

## Conclusion

This present study has further validated the use of CT scans with the Klales et al. [5] standard in a contemporary Indonesian sample. Its improved performance against the global function in Kenyhercz et al. [8] underscores the importance of population-specific models. When considered, age-dependent models showed misclassifications shift from females to males in older age groups, which support the assertion of pelvic traits becoming ‘masculinised’ as individuals age. Overall, this study provides forensic practitioners in Indonesia access to more morphoscopic sex estimation standards optimised for its population.
